# Are non-competitors greener? The effect of consumer awareness differences on green food consumption

**DOI:** 10.3389/fpsyg.2023.1276261

**Published:** 2023-12-04

**Authors:** Manhua Zheng, Qiujin Zheng, Jianhong Chen, Decong Tang

**Affiliations:** ^1^College of Economics and Management, Fujian Agriculture and Forestry University, Fuzhou, China; ^2^School of Journalism and Communication, Minjiang University, Fuzhou, China; ^3^College of Rural Revitalization, Fujian Agriculture and Forestry University, Fuzhou, China

**Keywords:** competitive awareness, environmental awareness, green self-efficacy, perceived control, green food purchase intention

## Abstract

**Introduction:**

Green consumption plays a crucial role in mitigating environmental degradation. Governments and corporations are actively fostering the growth of green consumption. The escalating environmental issues have awakened consumers' environmental and competitive awareness, which significantly aids in increasing the probability of green food consumption.

**Methods:**

This study, based on the Self-Consistency Theory and the Theory of Planned Behavior, constructs a model to analyze the effects of consumer competitive and environmental awareness on green food purchase intentions. Data from 700 consumer surveys were examined through structural equation modeling.

**Results:**

Findings indicate that while consumer competitive awareness negatively impacts green self-efficacy and perceived control, environmental awareness has a positive effect. Green self-efficacy and perceived control both positively influence green food purchase intentions. Notably, competitive awareness has a more substantial negative impact on perceived control compared to green self-efficacy. In contrast, the positive influence of environmental awareness on green self-efficacy is stronger than on perceived control. Moreover, the effect of green self-efficacy on green food purchase intention is more pronounced than that of perceived control.

**Discussion:**

Strategies like enhancing media publicity, educational initiatives, and improving purchase convenience can increase consumer purchase intentions. This study offers valuable insights for governments and businesses in understanding consumer psychology in green food consumption, aiding in marketing strategies for green food products.

## 1 Introduction

The deterioration of the ecological environment is a global challenge, reminiscent of the tragedy of the commons in economics. For governments, reducing environmental pollution and carbon emissions is a critical issue (Guo et al., 2022). Individually, environmental problems affect people's quality of life, leading to a shift in consumers' green consumption awareness and attitudes (Yam-Tang and Chan, 1998). Human activities, such as industrial emissions, transportation, agricultural activities, urban development, waste management, and consumption habits, are key drivers of many environmental issues (Schultz, 2011). Although studies have highlighted the impact of production and living behaviors on environmental problems, the specific mechanisms of improving environmental issues through personal consumption behaviors remain unclear.

Green food, defined by Zheng et al. (2022), refers to food produced in a healthy ecological environment, cultivated, processed, packaged, stored, and transported under strict standards with comprehensive quality control. It bears the green food label, meeting environmental, health, and social sustainability requirements, ensuring safety, quality, and pollution-free products. Promoting green food consumption can alleviate environmental problems caused by agricultural activities and food processing, ensuring consumer health. Similar to the tragedy of the commons, governments and businesses advocating green consumption can reduce environmental pollution from fertilizers and pesticides, lower carbon emissions, and decrease resource consumption. Promoting green consumption is key to achieving sustainable development for both the environment and businesses (Yan et al., 2021). For consumers, green food, compared to regular agricultural products, has lower levels of fertilizers, pesticides, and heavy metals, reducing the risk of food poisoning and ensuring health (Zheng et al., 2023). The concept of individual rights in the tragedy of the commons suggests that consumer choices can impact the health of public resources. Therefore, advocating for the consumption of green food is a way for individuals to contribute to the common good of society. However, the specifics of how consumer behavior can promote the prevalence of green food and environmental sustainability require further in-depth study.

The strength of consumer awareness can enhance the likelihood of green food consumption (Le et al., 2022). Environmental degradation can trigger two types of consumer awareness: first, a protective awareness toward the environment on which they depend (Janmaimool and Chudech, 2020). As concerns about climate change, water scarcity, and biodiversity loss grow, along with government advocacy, consumers are becoming increasingly aware of issues related to water sources, land pollution, and air quality. Motivated by their health and quality of life, they are inclined to purchase green foods. This is because green foods emphasize environmental protection during production, processing, and storage, thereby reducing pollution of air, land, and water resources. Second, a competitive awareness toward environmental resources (Fritze et al., 2008), as conceptualized in the tragedy of the commons, where public resources are overused or encroached upon competitively, leading to resource depletion. On one hand, higher consumer competitive awareness may reduce interest and motivation in purchasing green food. In contexts of environmental degradation and resource scarcity, competitive thinking is activated (Roux et al., 2015), with consumers focusing more on cost, price, and durability (Zhu et al., 2018), and neglecting the green attributes of products due to perceived poor cost-effectiveness. On the other hand, as consumer environmental awareness increases, more consumers start valuing the importance of green purchasing (Lin and Huang, 2012), focusing on food quality and safety, and recognizing the benefits of green food for health and the environment (Zheng et al., 2023). Current research primarily focuses on consumer cognition regarding environmental protection and resource competition, with the specific impact of consumer awareness on green food consumption behavior remaining unclear.

Research on the factors influencing green consumption willingness can be categorized into three parts. First, demographic characteristics, including gender, age, education level, etc. (Chekima et al., 2016; Alzubaidi et al., 2021), where studies find women are more likely to adopt green consumption behaviors, while marital status is unrelated to environmental attitudes (Diamantopoulos et al., 2003). Second, external environmental factors, including price, labeling, government, advertising, etc. (Zheng et al., 2022; Hu and Meng, 2023; Lu and Li, 2023). Third, psychological factors, including consumer attitudes, cognition, knowledge, and environmental concerns (Meinhold and Malkus, 2005; Pagiaslis and Krontalis, 2014; Zheng et al., 2023).

While these studies lay the foundation for research on green consumption behaviors, they fail to comprehensively analyze consumer behavior from the perspective of facing environmental and competitive challenges. Existing research lacks a comprehensive analysis of consumer awareness, especially from the perspective of competitive awareness, on green consumption behavior. This study aims to fill this research gap, exploring how competitive awareness and environmental awareness jointly influence consumers' green food purchase decisions. Scholarly research on competitive awareness has primarily focused on interpersonal behaviors, such as willingness to cooperate (Van Lange, 1999), willingness to contribute (Van Lange et al., 1997), and social comparison behaviors (Stapel and Koomen, 2005). Notably, both competitive awareness and environmental awareness play significant roles in green consumption willingness research. Competitive awareness can stimulate a competitive mindset in consumers, driving them toward green products to showcase their environmental concerns and social responsibility. Additionally, environmental awareness reflects consumers' understanding and cognition level of environmental issues, guiding the entire purchasing decision process, and thus influencing their consumption of green food.

This study aims to bridge this gap by constructing a theoretical model of the impact of consumer awareness (environmental and competitive awareness) on green food consumption. Based on the Self-Consistency Theory and the Theory of Planned Behavior (TPB), this research will delve into how consumers balance competitive awareness and environmental awareness in decision-making processes involving environmental and social responsibilities. This endeavor seeks to enrich research on green consumption behaviors, providing references for governments and businesses to further unleash the potential of green consumption.

## 2 Research hypotheses and theoretical framework

### 2.1 Research hypotheses

#### 2.1.1 Competitive awareness and green self-efficacy

Competitive awareness refers to an individual's desire and psychological inclination to achieve personal success, enhance potential, and fulfill personal goals (Roux et al., 2015). Green self-efficacy denotes an individual's confidence and capability to undertake pro-environmental actions, i.e., self-assessment of achieving environmental protection goals (Meinhold and Malkus, 2005). Consumers' competitive awareness can lead to a focus on immediate personal benefits while overlooking long-term environmental impacts, weakening their green self-efficacy.

In decision-making, competitive awareness may prompt excessive focus on personal gain, intensifying selfish behavior. In social contexts, competitive awareness might drive seemingly altruistic behavior, albeit still grounded in self-interest (Roux et al., 2015). Excessive competitive awareness can lead to egocentrism, neglecting others' needs, future personal requirements, and potential environmental impacts. This may result in consumers prioritizing personal gain over eco-friendliness and sustainability, potentially leading to unethical or self-serving behaviors (Van Lange, 1999). In this context, consumers may purchase unnecessary and low-quality goods, thereby exacerbating environmental burdens, while also neglecting their social responsibility regarding environmental issues. This leads to a decline in green self-efficacy.

For instance, panic buying triggered by the pandemic can have negative societal impacts (Chua et al., 2021). This situation fosters a competitive mindset among consumers over existing resources, leading to over-purchasing of goods and essentials. Such behavior results in resource wastage and can cause excessive greenhouse gas emissions (Pappalardo et al., 2020), intensifying environmental strain. In this scenario, consumers prioritize ensuring that the products they purchase meet sanitary standards, often overlooking their environmental impact. This focus can diminish their motivation to actively address environmental issues, subsequently reducing their green self-efficacy.

Based on this analysis, we propose:

**H1:** Consumer competitive awareness negatively impacts green self-efficacy.

#### 2.1.2 Competitive awareness and perceived control

Perceived control refers to the consumer's sense of having power and decision-making authority in their purchasing behavior. However, this perceived control can be influenced by a consumer's competitive awareness. Competitive awareness leads consumers to focus more on comparisons with others (Stapel and Koomen, 2005), resulting in increased self-awareness and hostility toward others, thereby reducing the spirit of cooperation (Van Lange et al., 1997). A strong competitive awareness can negatively impact the consumer's psychology, such as questioning their abilities or actions, leading to a decline in self-confidence and, consequently, reduced perceived control.

Under the influence of competitive awareness, consumers may make unsuitable consumption decisions. Time pressure (Dambacher et al., 2011) can lead to impulsive purchasing (Zhang and Zhang, 2022), neglecting real needs and quality, affecting perceived control. Additionally, competitive awareness can induce stress and anxiety during purchasing. This is because competitive awareness makes consumers perceive the scarcity of goods, leading them to believe in supply chain disruptions and instability in their surrounding environment, which increases their sense of loss of control (Bonneux and Van Damme, 2006; Chua et al., 2021).

In summary, competitive awareness can negatively impact consumers' perceived control. On one hand, it can weaken this perceived control by inducing impulsive buying and reducing self-confidence. On the other hand, competitive awareness can cause consumers to feel the instability of supply chains during the purchasing process, thus affecting their control over purchasing decisions.

Based on this analysis, we propose:

**H2:** Consumer competitive awareness negatively impacts perceived control.

#### 2.1.3 Environmental awareness and green self-efficacy

Consumer environmental awareness refers to the recognition and concern for environmental issues and the willingness to strive for ecological solutions (Dunlap and Jones, 2002). This awareness is not only reflected in the recognition of environmental issues but also in the attitude of consumers toward taking action to protect the environment. Green self-efficacy refers to an individual's confidence and sense of ability to engage in environmentally friendly behaviors (Meinhold and Malkus, 2005). Higher environmental awareness helps consumers understand green food and its environmental benefits (Lin and Chang, 2012), like standards and mechanisms, and the positive role in reducing environmental pollution, making consumers more willing to make efforts to alleviate ecological and environmental issues.

Consumers with strong environmental awareness are more likely to recognize the impact of their actions on the environment and engage in green behaviors, like choosing eco-friendly hotels, cars (Okada et al., 2019), and organic food (Basha et al., 2015). These behaviors not only reflect the consumers' attitude toward environmental protection but also demonstrate their ability to achieve green goals through practical actions. Therefore, these actions, in turn, enhance their sense of green self-efficacy, which is the recognition of their confidence and ability to carry out environmentally friendly behaviors.

Furthermore, the greater the attention to environmental issues, the higher the consumers' regard for ecological sustainability, leading to the formation of a more positive attitude toward environmental protection (Wang et al., 2020). Studies have shown that environmental awareness can positively influence attitudes, subjective norms, and perceived behavioral control (Xu et al., 2020). This heightened awareness helps consumers to gain a more comprehensive understanding of environmental knowledge and skills, thereby changing their mindset and perceptions toward adopting green technologies' cognitive values, i.e., from their attitudes (environmental awareness) to influencing their perceived efficiency of behavior (self-efficacy) (Jiang et al., 2022).

Based on this analysis, we propose:

**H3:** Consumer environmental awareness positively influences green self-efficacy.

#### 2.1.4 Environmental awareness and perceived control

According to the TPB, environmental awareness positively impacts consumers' perceived behavioral control (Xu et al., 2020). Consumers' negative environmental behaviors may stem from a lack of awareness about environmental issues. Increased environmental awareness can help consumers recognize their impact on the environment and contribute to its mitigation (Wang et al., 2023). When consumers are more aware and concerned about environmental issues, they are more capable of realizing that their purchasing behavior can impact the environment, thereby becoming more motivated to engage in green consumption (Slamet et al., 2016). This positive shift in behavior can enhance consumers' sense of self-control, enabling them to take environmentally friendly actions with greater confidence.

For consumers with high environmental awareness, their environmental knowledge and eco-friendly skills can further enhance their self-efficacy related to the environment. Consumers equipped with such abilities are more adept at obtaining and discerning information about green products, leading to more accurate purchasing decisions or contributing to environmental protection through actions like recycling and reuse (Ruangkanjanases et al., 2020). This enhancement of abilities helps to strengthen their control over purchasing behavior, making consumers feel effectively in charge of their actions, thus boosting their perceived control.

Based on this analysis, we propose:

**H4:** Consumer environmental awareness positively influences perceived control.

#### 2.1.5 Green self-efficacy and green food purchase intention

According to the Self-Consistency Theory, strong green self-efficacy, the belief in one's ability to engage in eco-friendly behavior, can translate into a stronger willingness to protect the environment (Hu and Meng, 2023), including purchasing green food, as consumers believe it benefits both personal health and the environment.

The Theory of Reasoned Action suggests that perceived efficacy in one's actions is a key predictor of behavioral intentions (Schifter and Ajzen, 1985). Studies have found that stronger perceived behavioral efficiency (e.g., farmers' self-efficacy) is closely linked to increased behavioral intentions (e.g., willingness for low-carbon production) (Jiang et al., 2022). Consumers with higher green self-efficacy, believing in their ability to achieve environmental goals through purchasing green food, are more likely to be motivated to consume green food.

After engaging in green consumption, if consumers gain positive experiences and effects from consuming green foods, such as improved health and increased environmental awareness (Zheng et al., 2023), this will enhance their willingness to continue purchasing green foods, thereby further boosting their intention to consume green foods. Related studies also indicate that green self-efficacy promotes the generation of pro-environmental behaviors in individuals (Jansson et al., 2010).

Based on this analysis, we propose:

**H5:** Consumer green self-efficacy positively influences green food purchase intention.

#### 2.1.6 Perceived control and green food purchase intention

Perceived behavioral control theory posits that an individual's perception of control significantly predicts their behavioral intentions (Adnan et al., 2019). Studies show that perceived behavioral control positively impacts the willingness to purchase green products (Xu et al., 2020). In that study, perceived behavioral control refers to the consumers' perception that they have the time, money, and ability to purchase green products, and the stronger this perceived control, the higher their behavioral intention (Wang et al., 2016). Perceived control can manifest as an assessment of the convenience of purchasing specific products (like recycled electronics), directly enhancing purchasing intentions (Yu et al., 2021). Perceived control, as a form of perceived behavioral control, represents an individual's assessment of resources and opportunities for specific behaviors, including consumers' sense of capability and obstacles in specific purchasing decisions (Choi and Park, 2017). When consumers feel effective control and decision-making in purchasing, they are more likely to lean toward green consumption. Conversely, difficulties or a sense of loss of control may hinder their engagement in eco-friendly actions (Wong et al., 2021).

Based on this analysis, we propose:

**H6:** Consumer perceived control positively influences the green food purchase intention.

### 2.2 Theoretical framework

Grounded in the Self-Consistency Theory, consumers are motivated toward products that align with their personal goals, which strengthens their psychological identification and actively encourages them to purchase that product (Sirgy, 1982). Specifically, when consumers' environmental awareness is effectively exhibited, it leads to consistent cognitive processing. This promotes a perspective that considers ecological aspects, stimulating green self-efficacy and belief in their ability to mitigate environmental issues, thereby adopting green consumption behaviors (Lin and Hsu, 2015).

According to the TPB, an individual's behavioral attitudes, subjective norms, and perceived behavioral control influence their behavioral intentions (Ajzen, 1991). In the context of green consumption, consumers' behavioral attitudes can be seen as their attitudes/awareness toward green consumption. Perceived behavioral control can be interpreted as consumers' perceived control and green self-efficacy, and individual behavioral intentions as consumers' green food purchase intention.

**Figure 1:**
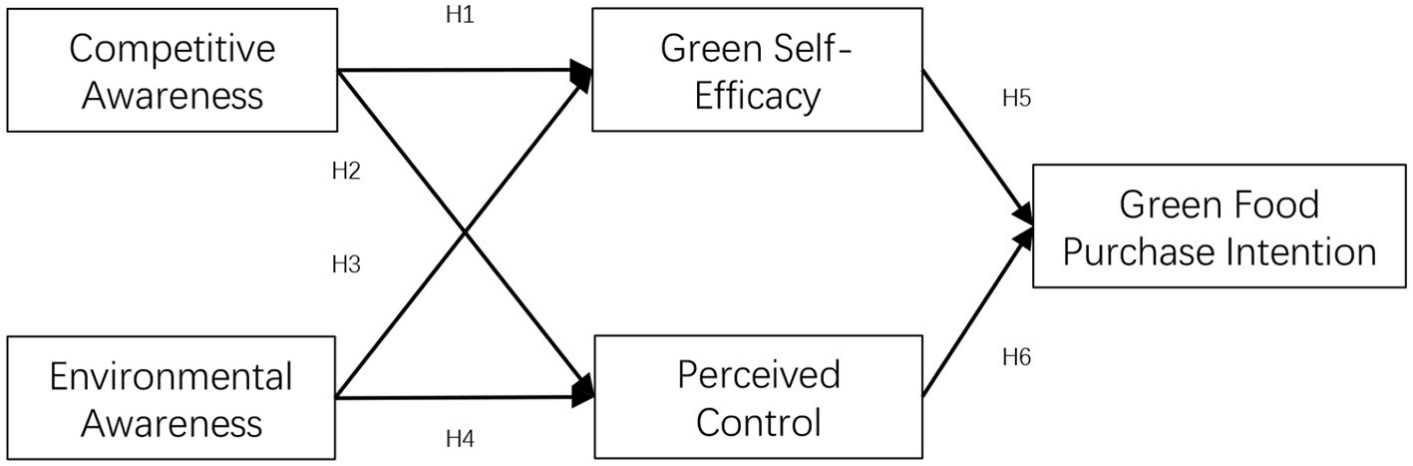
Theoretical model of the influence of consumer awareness on the green food purchase intention.

Based on the above analysis, a theoretical model of consumer awareness influencing green food purchase intention is constructed, as shown in Figure 1.

## 3 Methods

### 3.1 Questionnaire design

The research survey is divided into three parts: an introductory statement, a measurement of the main variables, and the respondents' basic information. First, the introduction primarily explains the purpose of the survey and assures confidentiality to alleviate any concerns of the respondents. Second, the measurement of the main variables includes five elements: competitive awareness, environmental awareness, green self-efficacy, perceived control, and green food purchase intention (the scales are shown in Table 1). The variables in this study were adapted from mature scales developed by previous scholars and measured using a 7-point Likert scale. The scale for competitive awareness is based on Ryckman et al. (1990)'s work, environmental awareness on Pagiaslis and Krontalis (2014), green self-efficacy on Chen et al. (2001), perceived control on Lachman and Weaver (1998), and green food purchase intention on Zheng et al. (2023). Third, the basic information section includes gender, age, education level, and income.

**Table 1:** Measurement items.

**Variables**	**Indicators**	**Items**	**References**
Competitive awareness (CA)	CA 1	I get jealous when my competitors are rewarded for their achievements	Ryckman et al., 1990
	CA 2	When I lose in a competition, I get sad	
	CA 3	I can't stand to lose in an argument	
	CA 4	Losing in the competition will make me feel inferior to others	
Environmental awareness (EA)	EA 1	I am concerned about environmental pollution	Pagiaslis and Krontalis, 2014
	EA 2	I am concerned about air and water pollution in the city	
	EA 3	I am concerned about the waste of water in the city	
	EA 4	I consider the impact on the environment when I buy products	
Green self-efficacy (GSE)	GSE 1	I think I can successfully practice the concept of environmental protection	Chen et al., 2001
	GSE 2	I feel I have the ability to help achieve environmental goals	
	GSE 3	I think I can effectively fulfill my environmental mission	
	GSE 4	I feel I am capable of dealing effectively with environmental issues	
	GSE 5	I think we can find creative solutions to environmental problems	
Perceived control (PC)	PC 1	Anything I'm determined to do, I can almost always do	Lachman and Weaver, 1998
	PC 2	If I really want to do something, I can usually find a way to succeed	
	PC 3	Whether I get what I want is within my control	
	PC 4	What my future will be depends mainly on me	
Green food purchase intention (GPI)	GPI 1	I consider buying green food	Zheng et al., 2023
	GPI 2	I am willing to buy green food if needed	
	GPI 3	Green food will excite me to buy	
	GPI 4	I think green food is worth buying	

### 3.2 Sample analysis

The study included two screening questions to ensure the reliability and attentiveness of the respondents. The first question, “Have you ever purchased green food?”, was used to identify valid samples, with only those responding “Yes” considered. The second, “Which of the following is a marine animal?”, was designed to ensure respondent attentiveness. In December 2022, the study distributed an online survey through the Credemo platform, widely recognized in the fields of consumer behavior and psychology. The platform first automatically excluded respondents who did not answer the second screening question seriously. Researchers then manually excluded invalid samples based on the first question. A total of 700 valid responses were collected, covering all provinces of China.

The demographic characteristics of the sample are shown in Table 2. The majority of the respondents were female (61.7%), aligning with the typical role of women in Chinese families as food purchasers and similar to previous green food research (Xu et al., 2022a). Most respondents were middle-aged and young adults (78.2%), which may be due to their deeper exposure to green food. A high level of education was observed (61.7% with a bachelor's degree or higher), possibly because green food companies find it less costly to disseminate information about green food to this demographic, who are more receptive to it, similar to previous findings (Zheng et al., 2023). The sample included a significant proportion of high-income earners, indicating the economic capability to purchase green food, consistent with the research objective and previous studies (Zheng et al., 2022).

**Table 2:** Sample characteristics.

**Item**	**Frequency**	**Percentage**
**Gender**		
Male	268	38.3%
Female	432	61.7%
**Age**		
18–22	98	14.0%
23–30	359	51.3%
31–40	188	26.9%
41–50	36	5.1%
Above 51	19	2.7%
**Education**		
High school or below	15	2.1%
Junior college	52	7.4%
Bachelor	520	74.3%
Master degree or above	113	16.1%
**Monthly income**		
4,000 RMB or below	155	22.1%
4,001–8,000 RMB	242	34.6%
8,001–12,000 RMB	202	28.9%
12,000 RMB or above	101	14.4%

## 4 Results

### 4.1 Reliability and validity analysis

The PLS-SEM analysis was conducted using SMARTPLS 3.0 software. The reliability results are presented in Table 3. The Cronbach's Alpha values for all five variables exceeded the critical threshold of 0.6, the Rho_A values were >0.7, and the Composite Reliability values surpassed 0.8. These results indicate that the scales demonstrate good internal consistency, affirming the reliability of the scales (Hair et al., 2011).

**Table 3:** Reliability and validity analysis.

**Constructs**	**Item**	**Loadings**	**Cronbach's α**	**Rho_A**	**CR**	**AVE**	**VIF**
CA	CA 1	0.832	0.840	0.864	0.891	0.673	1.779
		CA 2	0.838					1.878
		CA 3	0.733					1.676
		CA 4	0.874					2.226
EA	EA 1	0.820	0.792	0.797	0.865	0.616	1.741
		EA 2	0.745					1.472
		EA 3	0.780					1.628
		EA 4	0.792					1.554
GSE	GSE 1	0.781	0.842	0.845	0.887	0.612	1.682
		GSE 2	0.801					1.923
		GSE 3	0.790					1.749
		GSE 4	0.816					2.043
		GSE 5	0.721					1.540
PC	PC 1	0.842	0.832	0.833	0.888	0.665	2.089
		PC 2	0.832					1.878
		PC 3	0.827					1.937
		PC 4	0.759					1.512
GPI	GPI 1	0.795	0.809	0.817	0.874	0.634	1.733
		GPI 2	0.771					1.666
		GPI 3	0.802					1.577
		GPI 4	0.818					1.712

Discriminant validity refers to the degree to which a variable is distinct from other variables. The study employed the Fornell-Larcker criterion to assess discriminant validity. According to Fornell and Larcker (1981) standards, the correlation coefficients between dimensions should be less than the Average Variance Extracted (AVE), meaning the values below the diagonal should be smaller than those on the diagonal. The discriminant validity of the study, as shown in Table 4, meets the Fornell-Larcker criterion, indicating good discriminant validity.

**Table 4:** Discriminant validity (FORNELL).

	**(1)**	**(2)**	**(3)**	**(4)**	**(5)**
Competitive awareness (1)	0.821				
Environmental awareness (2)	−0.219	0.785			
Green self-efficacy (3)	−0.312	0.724	0.783		
Perceived control (4)	−0.346	0.499	0.638	0.816	
Green food purchase intention (5)	−0.153	0.661	0.622	0.481	0.796

### 4.2 Assessment of structural model

The PLS-SEM analysis was performed using SMARTPLS 3.0 software. Initially, the PLS algorithm was employed to calculate the path coefficient values for each relationship, followed by the application of Bootstrapping to assess the significance of these path coefficients, with a subsample size set to 5,000. Path coefficients are considered significant when the *t*-value exceeds 1.96, and the *p*-value is < 0.05. The research results, presented in Table 5, indicate that all hypotheses were supported, with corresponding path coefficients meeting the specified criteria.

**Table 5:** Model path coefficients.

	**β**	**Mean**	**SD**	***t*-value**	***p*-value**	**95% LLCI**	**95% ULCI**	**Decision**
CA ->GSE	−0.161	−0.161	0.023	7.100	0.000	−0.199	−0.123	Supported
CA ->PC	−0.248	−0.249	0.029	8.460	0.000	−0.297	−0.200	Supported
EA ->GSE	0.689	0.689	0.026	26.075	0.000	0.644	0.731	Supported
EA ->PC	0.445	0.446	0.036	12.411	0.000	0.386	0.505	Supported
GSE ->GPI	0.532	0.535	0.061	8.732	0.000	0.434	0.632	Supported
PC ->GPI	0.142	0.140	0.063	2.272	0.023	0.037	0.243	Supported

**Figure 2:**
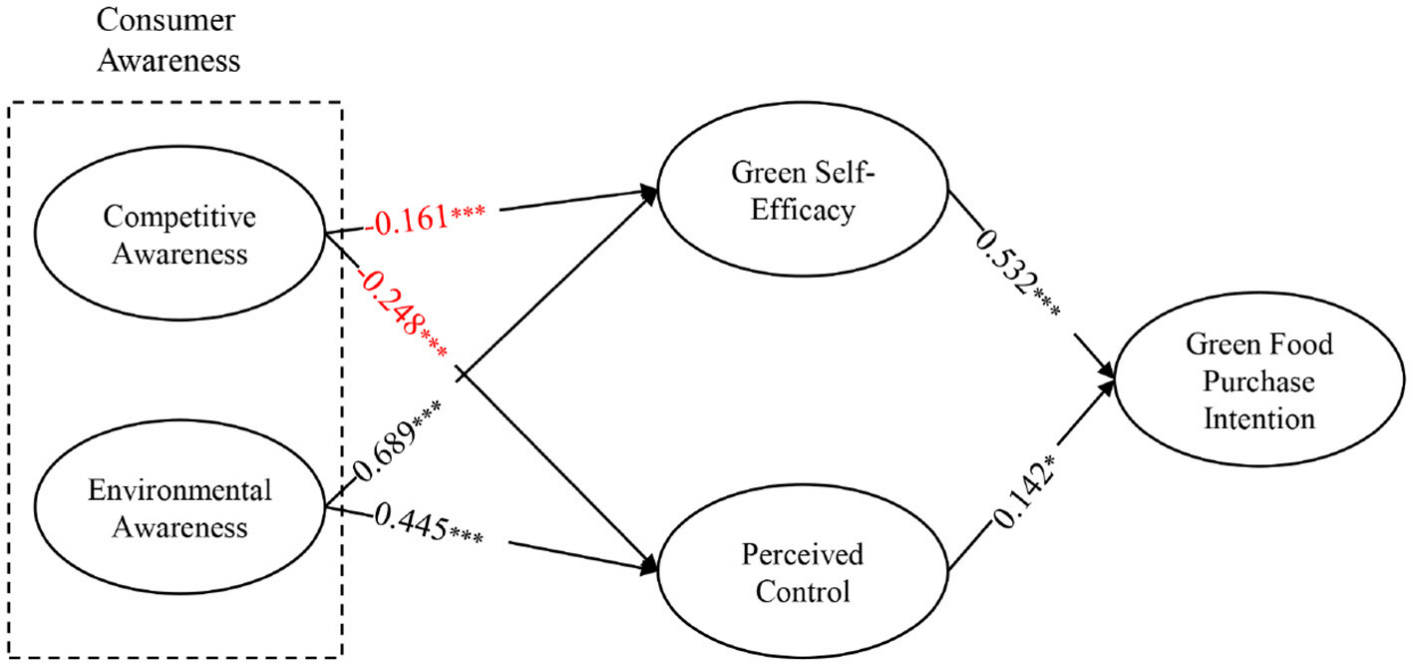
Structural equation modeling. *means *p* < 0.05 and ***means *p* < 0.001.

From the perspective of path coefficients (Figure 2), the negative impact of competitive awareness on the perceived control is greater than its impact on green self-efficacy. The positive influence of environmental awareness on green self-efficacy exceeds its impact on perceived control. Additionally, the positive effect of green self-efficacy on the willingness to consume green products is greater than that of the perceived control.

### 4.3 Indirect effects testing

The study analyzed indirect and mediating effects through the assessment of total indirect effects and specific indirect effects. The results are depicted in Tables 6, 7. The findings show that all indirect effects are significant, with *t*-values exceeding 1.96 and *p*-values below 0.05.

**Table 6:** Total indirect effect.

	**β**	**Mean**	**SD**	***t*-value**	***p*-value**
CA->GPI	−0.121	−0.121	0.016	7.497	0.000
EA ->GPI	0.430	0.432	0.033	13.111	0.000

**Table 7:** Specific indirect effects.

	**β**	**Mean**	**SD**	***t*-value**	***p*-value**
CA -> GSE -> GPI	−0.085	−0.086	0.015	5.664	0.000
CA -> PC-> GPI	−0.035	−0.035	0.016	2.218	0.027
EA -> GSE -> GPI	0.366	0.370	0.051	7.132	0.000
EA -> PC-> GPI	0.063	0.063	0.030	2.143	0.032

## 5 Research conclusions and management implications

### 5.1 Conclusions

The study, grounded in the Self-Consistency Theory and the Theory of Planned Behavior, constructs a model examining the impact of consumer competitive awareness and environmental awareness on the intention to consume green food. Using a sample of 700 green food consumers and employing structural equation modeling for analysis, it was found that consumer competitive awareness negatively impacts green self-efficacy and perceived control. Conversely, consumer environmental awareness positively influences green self-efficacy and perceived control. Moreover, both green self-efficacy and perceived control positively affect the intention to consume green food.

### 5.2 Discussion

The finding that competitive awareness significantly negatively impacts green self-efficacy and perceived control differs from previous research, which primarily focused on panic buying behavior (Singh et al., 2023), aggressive behavior (Stapel and Koomen, 2005), and self-interest actions (Roux et al., 2015), or viewed competitive awareness mainly as a moderating factor (Song et al., 2021). Few studies have delved into how competitive awareness affects green self-efficacy and perceived control, possibly due to its complex psychological nature involving cognition, self, and adaptability (Mildawani et al., 2022). This study uniquely analyzes the consumer awareness sub-dimension - competitive awareness from the perspective of negative resource competition caused by environmental issues.

The significant positive impact of environmental awareness on green self-efficacy and perceived control also differs from previous research, which mainly focused on direct effects like purchasing intentions (Hao and Chenyue, 2021), learning strategies (Newton et al., 2015), environmental attitudes (Leclercq-Machado et al., 2022), and willingness to pay a premium (Konuk, 2018). There has been limited exploration into how environmental awareness influences green self-efficacy and perceived control. The impact mechanism varies due to the differing interpretations of environmental awareness (also termed ecological awareness, green awareness, environmental literacy, and environmental attitudes) in consumer behavior.

The study also finds that green self-efficacy and perceived control significantly positively influence green food consumption intentions, diverging from previous research focusing on personal characteristics, consumer cognition, attention, trust, motivation, and purchasing atmosphere (Alzubaidi et al., 2021; Ma et al., 2022; Zheng et al., 2023). There are relatively few studies focusing on how green self-efficacy and perceived control significantly influence the intention to consume green food.

Moreover, studies have shown that perceived behavioral control does not significantly affect low-carbon product purchasing intentions (Li et al., 2017), contrasting with our findings, possibly due to different decisive factors in purchasing various products and regional differences (Yu et al., 2021).

### 5.3 Theoretical contributions

First, the study categorizes consumer awareness into environmental and competitive awareness awareness, enhancing the scope of consumer awareness research. Previous studies primarily analyzed consumer altruism and self-interest, categorizing it into environmental, component, health, and food safety awareness (Bornkessel et al., 2014; Hojnik et al., 2019; Xu et al., 2022b), with different environmental awareness leading to varied green product preferences (Kanchanapibul et al., 2014). These studies primarily analyze the direct impact of consumer positive awareness on consumption from the perspective of consumer awareness (Hwang, 2016) or focus on a single dimension in analyzing consumer behavior. There is less emphasis on examining the indirect effects of consumer awareness from different dimensions. This paper, by incorporating competitive awareness from a negative perspective of consumers, further refines consumer awareness theories and models, enhancing their explanatory power. It adds an explanation of the negative effects of green consumption, providing a more comprehensive understanding of consumer behavior in this context.

Second, the study provides a new research model for the impact of different dimensions of consumer awareness on consumption intentions and enriches the research on mediating variables between consumer awareness and consumption intentions. Previous mediator variable research included product familiarity, environmental responsibility, attitudes, subjective norms, perceived behavioral control, learning strategies, etc. (Newton et al., 2015; Hojnik et al., 2019; Zhang and Luo, 2021). The mechanisms of influence in this area exhibit a certain complexity and lack a unified understanding. Additionally, there is a scarcity of research focusing on green self-efficacy and perceived control as mediating variables. While some studies have analyzed the impact of self-efficacy on consumption intentions, these have primarily been conducted from perspectives such as self-monitoring, self-esteem, personal preferences, financial scarcity, anxiety, digital literacy, and other similar aspects (Lin and Hsu, 2015; Hu and Meng, 2023; Zhang et al., 2023). Limited research has analyzed the impact of perceived control on consumption intentions. However, these studies have primarily approached the topic from perspectives such as perceived risk, the severity of pandemics, crisis of product hazards in industries, perceived economic liquidity, and socioeconomic status (Yoon and Kim, 2018; Li et al., 2019; Zhang and Luo, 2021).

Additionally, some research suggests environmental awareness is challenging to translate into consumer behavior, or it only plays a moderating role in consumption intentions (De Canio et al., 2021). This study delves into the impact mechanisms of consumer awareness, green self-efficacy, perceived control, and green consumption intentions, highlighting the strong mediating role of green self-efficacy and its importance in influencing consumer purchasing decisions, enriching the research on green self-efficacy.

### 5.4 Management implications

#### 5.4.1 Increase awareness through media and educational programs

Begin with school education, enhancing the dissemination of green environmental knowledge through textbooks, conferences, and educational campaigns, cultivating environmental awareness among students and teachers.

On one hand, efforts can be made to strengthen consumer education about environmental issues through various channels such as media (TV ads, Weibo, TikTok, Bilibili, promotional brochures, etc.), communities, and environmental organizations. This would help consumers become aware of the current environmental situation, like soil and water pollution, and realize the importance and urgency of protecting the environment. On the other hand, media can be used to inform consumers about the characteristics, production methods, transportation, labeling, and benefits of green foods. Enhancing consumer knowledge and understanding of the environmental benefits of green foods can make them more aware of the impact their green food consumption has on the environment and society, thereby increasing their perceived control.

#### 5.4.2 Reduce consumer competitive awareness by improving purchase convenience and comfort

Enhance the convenience of purchasing green food. Businesses can increase sales channels for green food (online official stores, individual broadcasters, offline supermarkets, convenience stores), expand sales scope, and diversify product categories (primary green food, processed green food—green dried fruits and vegetables, gluten-free green food, low-fat green food, etc.), making it easier for consumers to access various types of green food and reducing the likelihood of choosing ordinary food due to competition.

Create a relaxing and enjoyable marketing atmosphere for consumers through the harmonious and comfortable design of online green food platforms, the selection of broadcasters, and the creation of self-service shared offline stores, reducing consumers' competitive mentality.

#### 5.4.3 Enhance consumer control through communication forums and green food recognition mechanisms

Green food companies can establish forums for green food discussions, providing channels for consumer feedback (such as tea forums, and academic discussion forums), allowing consumers to learn about the benefits of green food (product quality, safety, health benefits), and encouraging purchases.

Build green food recognition mechanisms, allowing consumers to adopt green food cultivation areas and choose whether to participate in offline planting and sales or cloud adoption and involvement in green food planting or sales. This provides consumers with opportunities to join green food production and sales organizations, allowing them to participate more directly in the production and sales of green food and enhance their perceived control.

#### 5.4.4 Enhance green self-efficacy through experiential activities, consumption guides, and role models

Organize experiential activities and publish guides for green food consumption to help consumers build confidence in green consumption, thereby improving their green self-efficacy.

Promoting environmental public welfare activities, as well as businesses and individuals who have contributed to environmental protection, can serve as role models to inspire consumers to choose green foods. Making consumers aware of those with a good reputation and positive word-of-mouth in the environmental field can increase trust in green foods through the demonstration effect, thereby enhancing green self-efficacy. Highlighting these positive examples can effectively motivate consumers by showing the tangible impact of choosing environmentally friendly options and recognizing the efforts of those leading the way in sustainable practices.

### 5.5 Research limitations and future directions

Limitations of the study sample. Since the survey was conducted in a single month in China, there may be national economic and cultural differences, as well as quarterly consumption variations. Future research could explore green food consumption from different national perspectives, cultural viewpoints, quarterly aspects, or before and after government policy implementation.

Boundary Conditions Undiscussed. The study did not discuss the moderating variables affecting green consumption. Currently, there are many influencing factors, and boundary conditions have not been further discussed. Current research primarily focuses on individual-level variables. Future studies could explore boundary conditions of green food consumption behavior from group-level (group pressure) and organizational-level (environmental organization consumption habits) perspectives.

## Data availability statement

The raw data supporting the conclusions of this article will be made available by the authors, without undue reservation.

## Ethics statement

Ethical review and approval was not required for the study of animals/human participants in accordance with the local legislation and institutional requirements. Written informed consent from the patients/ participants or patients/participants legal guardian/next of kin was not required to participate in this study in accordance with the national legislation and the institutional requirements.

## Author contributions

MZ: Conceptualization, Methodology, Writing—original draft. QZ: Resources, Supervision, Writing—review & editing. JC: Writing—review & editing. DT: Formal analysis, Supervision, Writing—review & editing.
